# Compositional study of Ti–Nb oxide (TiNb_2_O_7_, Ti_2_Nb_10_O_29_, Ti_2_Nb_14_O_39_, and TiNb_24_O_62_) anodes for high power Li ion batteries†

**DOI:** 10.1039/d4ta08141b

**Published:** 2025-02-19

**Authors:** Yeonguk Son, Haeseong Jang, Bo Wen, Changshin Jo, Alexander S. Groombridge, Adam Boies, Min Gyu Kim, Michael De Volder

**Affiliations:** a Department of Engineering, University of Cambridge 17 Charles Babbage Road CB3 0FS Cambridge UK mfld2@cam.ac.uk; b Department of Chemical Engineering, Changwon National University Changwon Gyeongsangnam-do 51140 Republic of Korea; c Department of Advanced Materials Engineering, Chung-Ang University 4726, Seodong-daero, Daedeok-myeon Anseong Gyeonggi-do 17546 Republic of Korea; d Department of Battery Engineering and Department of Chemical Engineering, Pohang University of Science and Technology University Pohang 37666 Republic of Korea; e Echion Technologies Ltd Unit 9, Cambridge South, West Way CB22 3FG Cambridge UK; f Department of Mechanical Engineering, Stanford University Stanford CA 94305 USA; g Beamline Research Division, Pohang Accelerator Laboratory (PAL) Pohang 37673 Republic of Korea habga82@postech.ac.kr

## Abstract

Titanium niobium oxides (TNOs) are attractive anode materials for high power density Li-ion batteries. However, the details of capacity storage in TNOs are not fully understood today as it depends on the Ti and Nb composition and their changes in the oxidation state. This is further complicated by a wide variation in gravimetric capacities reported in the literature for TNO anodes. Therefore, in this work, we systematically synthesise TiNb_2_O_7_, Ti_2_Nb_10_O_29_, Ti_2_Nb_14_O_39_, and TiNb_24_O_62_ particles using the same solid state reaction approach and report their electrochemical properties *via* galvanostatic cycling, cyclic voltammetry, and the galvanostatic intermittent titration technique (GITT). Furthermore, we use *operando* X-ray absorption spectroscopy (XAS) to investigate the redox reactions taking place in each of these compositions, which provides new insights into their charge storage mechanisms. We found that of the materials tested, TiNb_2_O_7_ anodes show the best cycling and rate performance, which could be related to the higher utilization of Nb redox revealed *via operando* XAS analysis.

## Abstract 2

Systematic synthesis of titanium niobium oxides unveils TiNb_2_O_7_'s superior cycling and rate performance, attributed to efficient Nb redox utilization, offering advancements in high-power Li-ion battery anodes.

## Graphical abstract

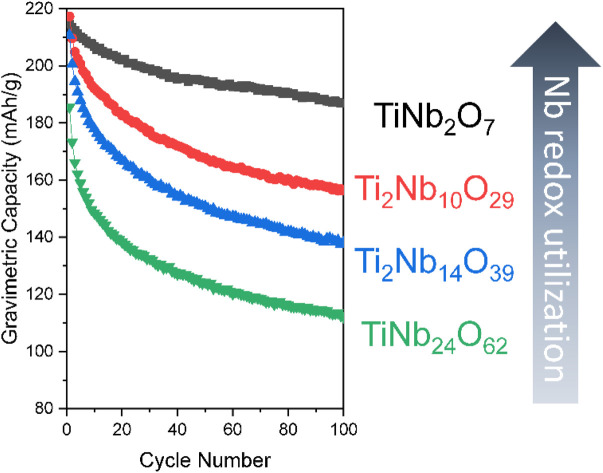

## Introduction

Lithium titanium oxide (LTO) is a commercially used anode material for high power lithium-ion batteries (LIBs).^1^ Although the gravimetric capacity (∼175 mA h g^−1^) and nominal voltage of LTO (∼1.55 V) are worse than those of commercial graphite anodes (360 mA h g^−1^ and 0.1 V respectively), LTO anodes show a superior rate performance, which is favourable for high power application.^1^ Hence, there have been many publications showing LTO anodes with great capacity retention, even at 10C-rates with high material areal loadings.^2^ In contrast, graphite anodes are typically limited in rate performance, which is in part due to its very low nominal voltage *versus* Li, which can lead to Li plating. The development of new LIB anodes for electric vehicles (EVs) needs to accommodate the requirements for a long driving range with those of short battery charging times and battery safety. It is therefore important to balance parameters such as the anode capacity, nominal voltage, rate performance and cost judiciously. LTO anodes were used in EVs such as Mitsubishi's i-MiEV and Honda's Fit EV, as well as power tools, despite their low capacity and high nominal voltage, as described above.^1^ However, most EVs have graphite anodes, and many manufacturers are exploring alternative materials that combine the rate performance of LTO with the capacity of graphite, which are promising for improving future EV batteries. Recently, titanium niobium oxide (TNO) has gained interest as one such material.^3–7^

The gravimetric capacity of reported TNO anodes ranges from 210 to 341 mA h g^−1^ depending on the material design and cut-off voltage, which is 20–95% higher than that of LTO anodes, while maintaining fast charging properties.^4,8^ However, the exact details of the redox mechanisms contributing to the gravimetric capacity measure in TNO anodes remain ambiguous. For example, Dr S. Dai and co-workers reported valence state variation of Ti and Nb during the initial discharge using *in situ* Ti K-edge and Nb K-edge X-ray absorption near edge structure (XANES) spectra in the 1.0 V and 3.0 V voltage range in TiNb_2_O_7_.^9^ As a result, Ti^4+^ and Nb^5+^ were reduced to Ti^3.2+^ and Nb^3.6+^, which well matched with an experimental discharge capacity of 281 mA h g^−1^. However, this could not explain the broad range of capacities reported in publications. Moreover, there are only a few reports investigating how redox mechanisms vary depending on the TNO formulation.^10,11^ In this work, we investigate the reaction mechanisms taking place in TNO anodes by systematically synthesising materials with different compositions (TiNb_2_O_7_, Ti_2_Nb_10_O_29_, Ti_2_Nb_14_O_39_, and TiNb_24_O_62_) using the same synthesis protocol and benchmarking them against LTO anodes. We developed a dry solid state synthesis method for the above four TNO formulations which allows for a side-by-side comparison of their capacity, rate performance, capacity retention and capacitive behavior. Furthermore, in order to study the charge storage mechanisms taking place in these materials, detailed electrochemical tests are carried out together with *operando* XAS to track the changes in the redox state of Ti and Nb as a function of the state of charge. These new fundamental insights into the operation of different TNO batteries are critical for their further optimisation and potential use in future EVs and other commercial applications.

## Results and discussion

**Fig. 1:**
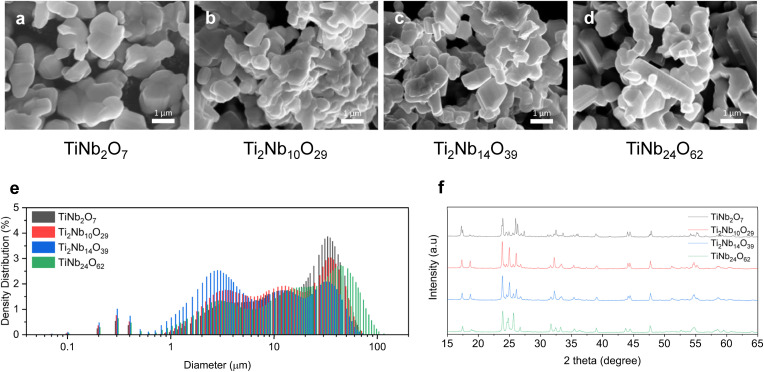
Morphology and crystalline structures of the as-prepared TNOs: SEM images of (a) TiNb_2_O_7_, (b) Ti_2_Nb_10_O_29_, (c) Ti_2_Nb_14_O_39_, and (d) TiNb_24_O_62_, (e) PSD results, and (f) powder XRD patterns of TNOs.

We synthesized 4 different compositions of TNO (TiNb_2_O_7_, Ti_2_Nb_10_O_29_, Ti_2_Nb_14_O_39_, and TiNb_24_O_62_) *via* solid state reactions. We blended TiO_2_ and NbO_2_ powders with a blade mixer and calcined them at 1000 °C under an oxygen atmosphere. A detailed description of the synthesis process is provided in the experimental section. The resulting shape and size of the four TNO compositions are shown in Fig. 1 and S1† and show a similar spread of diameters, which therefore allows for a fair direct comparison of their electrochemical performance. The crystalline structure of TNO materials is based on ReO_3_-type crystal building blocks, which are formed by corner- and/or edge-sharing octahedra and a small number of tetrahedra.^10^ To verify the crystalline structure of the 4 different compositions of TNO, XRD analysis and Rietveld refinement were carried out (Fig. 1f and S2†). The Rietveld refinement results shown in Fig. S2† match with previous reports, which validates the synthesis protocol developed in this work.^10,12–14^ Details of the refined lattice parameters are summarized in Table S1.†^9,15,16^ TiNb_2_O_7_, Ti_2_Nb_10_O_29_, and TiNb_24_O_62_ are constructed with structural units of a corner- and edge-shared 3 × 3 octahedron block (space group *C*2/*m*), 3 × 4 octahedron block (space group *A*2/*m*), and 3 × 4 octahedron block plus 0.5 tetrahedron at the block corner (space group *C*2) respectively.^17^ All the other compositions of Ti–Nb oxides studied here have a single phase except TiNb_2_O_7_ which has a mixed phase of TiNb_2_O_7_ (82.04%) and H phase Nb_2_O_5_ (17.96%). Ti and Nb are homogeneously mixed in the Ti–Nb oxide structures because the ionic radii of Ti^4+^ and Nb^5+^ are similar (0.61 Å for Ti^4+^ and 0.64 Å for Nb^5+^).^18^

The energy storage in the TNO anode is driven by changes in oxidation states of Ti and Nb during charge and discharge. According to previous reports, Ti^4+^ and Nb^5+^ are converted to Ti^3+^ and Nb^3+^ during the charging (lithiation) process.^10^ The theoretical capacities of TNO anodes depend on the composition of Ti and Nb and can be calculated by assuming a certain oxidation state change and normalising the charges stored per unit mass. We carried out these calculations (see the ESI† for details) under different assumptions:

(i) Ti^4+^ and Nb^5+^ are converted entirely to Ti^3+^ and Nb^3+^ (one-electron transfer in Ti and two-electron transfer in Nb): this results in theoretical capacities of 388 mA h g^−1^ for TiNb_2_O_7_, of 396 mA h g^−1^ for Ti_2_Nb_10_O_29_, of 398 mA h g^−1^ for Ti_2_Nb_14_O_39_, and of 402 mA h g^−1^ for TiNb_24_O_62_.

(ii) Ti^4+^ and Nb^5+^ are converted to Ti^3+^ and Nb^4+^ (one-electron transfer in Ti and one-electron transfer in Nb): this results in theoretical capacities of 233 mA h g^−1^ for TiNb_2_O_7_, of 216 mA h g^−1^ for Ti_2_Nb_10_O_29_, of 212 mA h g^−1^ for Ti_2_Nb_14_O_39_, and of 205 mA h g^−1^ for TiNb_24_O_62_.

These calculations illustrate that the capacity of TNO anodes can vary substantially depending on the actual oxidation state changes that are achieved in Ti and Nb within the voltage window they are cycled. It is worth noting that the capacities of the TNO anode reported in previous publications vary broadly (210–326 mA h g^−1^) and we summarized these values in Table S2.†^3,8,11,19–27^

**Fig. 2:**
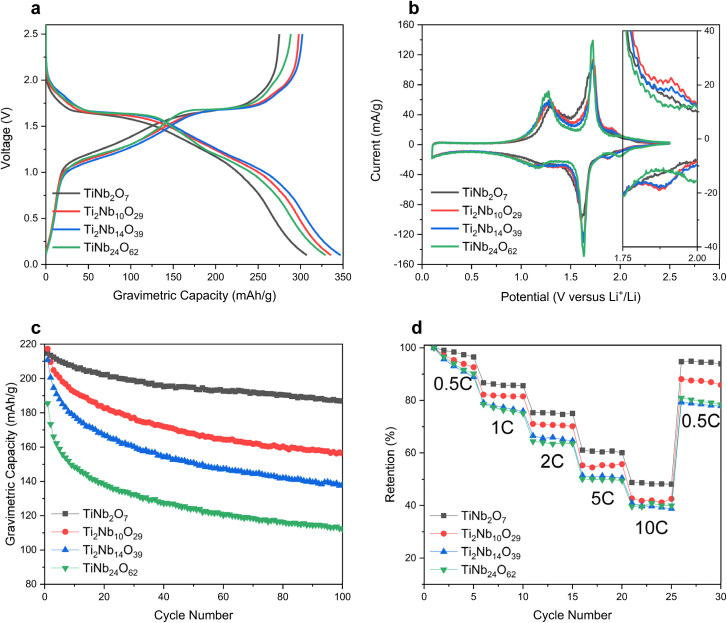
Electrochemical performance of the as-prepared TNO anodes: (a) 0.05C first formation voltage profiles of the TNO anodes, (b) 0.04 mV s^−1^ cyclic voltammetry (inset: magnified part from 1.75 to 2.00 V), and (c) 0.5C cycling performances of the TNO anodes. (d) Rate performances of the TNO anodes.

We first carried out half-cell experiments in coin cells with active material : carboxymethyl cellulose (CMC)/styrene butadiene rubber (SBR) binder : Super-P carbon additive at a weight ratio of 8 : 1 : 1 and tested them in triplicate. All electrodes were coated on Cu foil with an areal loading of 1.5–2.0 mg cm^−2^ and were tested using 1.3 M LiPF_6_ in a mixture of ethylene carbonate (EC), ethyl methyl carbonate (EMC) and diethyl carbonate (DEC) (3 : 5 : 2) with 10 wt% fluoroethylene carbonate (FEC) as an electrolyte. Cut-off voltage was 0.1–2.5 V. Our TiNb_2_O_7_, Ti_2_Nb_10_O_29_, Ti_2_Nb_14_O_39_, and TiNb_24_O_62_ anodes show reversible capacities of 278, 299, 302, and 289 mA h g^−1^ respectively and coulombic efficiencies of 88.1, 88.7, 87.7 and 87.5% in the first 0.05C formation cycle (Fig. 2a and S3†). The capacities measured fall between the two different oxidation state assumptions made above, and this warrants a more detailed investigation of the actual changes in oxidation taking place. To confirm the extent and potentials of each redox reaction, cyclic voltammetry (CV) was performed at a slow scan rate of 0.04 mV s^−1^ (Fig. 2b). The major lithiation and delithiation peaks of all the TNO compositions studied here are between 1.6 and 1.7 V *vs.* Li/Li^+^ respectively, which has previously been associated with the redox reactions of Nb^5+^/Nb^4+^.^28,29^ The second highest redox peak couple appears at around 1.1–1.3 V (lithiation) and 1.3 V (delithiation), which has been linked to the redox reactions of Nb^4+^/Nb^3+^. The lithiation peak of Nb^4+^/Nb^3+^ was broader than the delithiation peak of Nb^4+^/Nb^3+^. The minor peak couples at 1.8–2.0 V are related to the redox reactions of Ti^4+^/Ti^3+^.^19,26,30^ The redox peaks of Ti^4+^/Ti^3+^ at the TiNb_24_O_62_ sample are at around 2.0 V and the redox peaks of Ti^4+^/Ti^3+^ at Ti_2_Nb_10_O_29_ and Ti_2_Nb_14_O_39_ appear at around 1.9 V (see the inset of Fig. 2b). However, TiNb_2_O_7_ shows no minor peaks at 1.8–2.0 V.

Fig. 2c shows cycling performance of TNO anodes at 0.5C in half-cells. Of the TNO anodes studied here, the TiNb_2_O_7_ anode showed the best cycling performance of 87% retention in the 100^th^ cycle. The Ti_2_Nb_10_O_29_, Ti_2_Nb_14_O_39_, and TiNb_24_O_62_ anodes show retentions of 72, 66, and 60% in the 100^th^ cycle respectively. TiNb_2_O_7_ anodes also show a better rate performance than the other TNO anodes studied here. As shown in Fig. 2b, TiNb_2_O_7_ achieved a capacity retention of 48% at 10C whereas Ti_2_Nb_10_O_29_, Ti_2_Nb_14_O_39_ and TiNb_24_O_62_ achieved about 42, 40 and 41% respectively. Note that in high-rate performance, slight differences in particle size may have an effect. However, in our experiments, the differences in electrochemical performance based on the Ti-to-Nb ratio in TNO anodes were not overshadowed by variations in particle size.

**Fig. 3:**
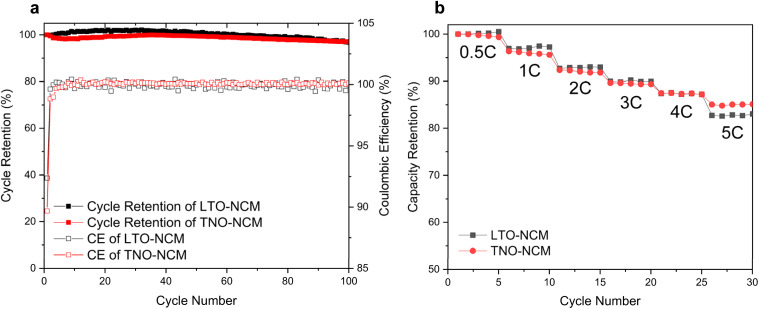
Full cell performance with TNO and LTO anodes. (a) Cycling performance of LTO–NCM and TNO–NCM full cells. (b) Rate performance of LTO–NCM and TNO–NCM full cells.

To verify the practical viability of our TNO anodes and to compare them with commercial LTO anodes, we compared the full cell performances of TNO and LTO anodes with the same LiNi_0.6_Co_0.2_Mn_0.2_O_2_ (NCM622) cathode with an N/P ratio of 1.4. We utilized the TiNb_2_O_7_ composition, which showed the best performance in our TNO anode half-cell tests, for the full cell experiments. Fig. S4† shows voltage profiles of full cell formation cycles. Gravimetric capacity of the full cell is based on the weight sum of both cathode and anode active materials. The discharge capacities and coulombic efficiencies of TNO–NCM and LTO–NCM full cells at the 1^st^ formation were 81.86 mA h g^−1^ and 91.41% (for TNO–NCM) and 74.13 mA h g^−1^ and 91.69% (for LTO–NCM) respectively. Nominal voltages of TNO–NCM and LTO–NCM full cells at the 1^st^ discharge were 2.19 and 2.29 V respectively. The nominal voltage of TNO–NCM is slightly lower than that of LTO–NCM; however the gravimetric capacity is slightly higher. The cycling performances of both TNO–NCM and LTO–NCM full cells showed very stable 0.5C cycling life (Fig. 3a). There is no significant fading in cycling life. Also, the two full cells show great rate performance at 0.5C charge and 0.5, 1, 2, 3, 4, and 5C discharge rates. At a 5C discharge rate, TNO–NCM and LTO–NCM full cells show a similar capacity retention of 85 and 83% respectively (Fig. 3b).

**Fig. 4:**
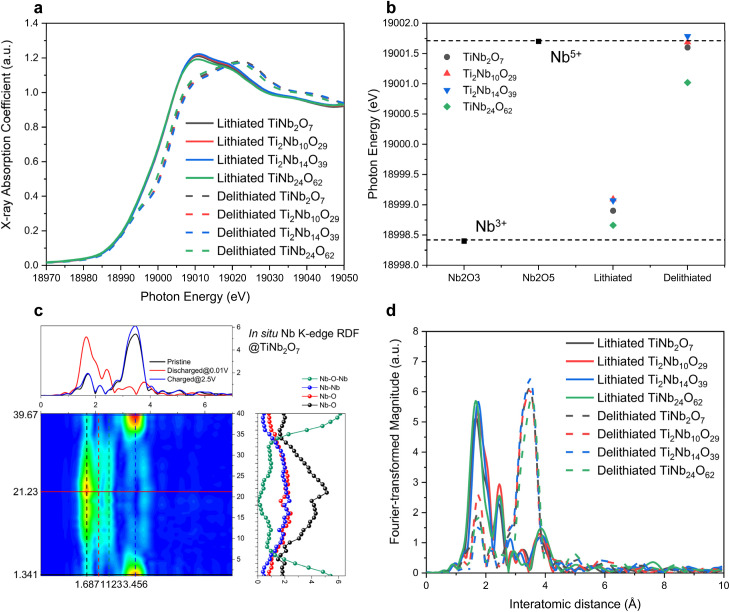
XANES and EXAFS analysis of the TNO anodes. (a) The Nb K-edge XANES absorption spectra of TNO anodes at fully lithiated and delithiated states, (b) the oxidation number of Nb at the TNO anode, (c) EXAFS results of TiNb_2_O_7_, and (d) Fourier transforms of the Nb K-edge EXAFS oscillations at fully lithiated and delithiated states.

To measure the oxidation state changes in Nb directly, we used *operando* XANES. Because of Ti's low threshold energy, *operando* XANES analysis is impossible because the photon energy corresponding to the Ti K-edge is almost entirely absorbed by the thick Cu foil. However, by measuring the actual oxidation state change of Nb with XANES alongside the capacity, we can calculate the capacity contribution from Ti and infer its oxidation state change (see the ESI† for the details of the calculation method). Fig. 4a shows the Nb K-edge XANES absorption spectra of TNO anodes at fully lithiated and delithiated states and continuous spectral changes are provided in Fig. S5.† Based on the results of XANES spectra and reference data, the oxidation number of Nb at the TNO anode is obtained from a least-squares method (LSM) (see Fig. 4b). Note that we utilized Nb_2_O_3_ and Nb_2_O_5_ as Nb^3+^ and Nb^5+^ reference materials respectively in oxidation state measurement in XANES analysis.^9^ During the delithiation process, the oxidation state changes of Nb in TiNb_2_O_7_, Ti_2_Nb_10_O_29_, Ti_2_Nb_14_O_39_, and TiNb_24_O_62_ electrodes are 1.64, 1.58, 1.59, and 1.43 respectively. The lower the ratio of Nb in TNO composition, the greater the electron transfer in Nb. In other words, the redox reaction of Nb is the most pronounced in TiNb_2_O_7_ and decreases with materials having a relatively higher Nb content. The calculated oxidation state changes of Ti at TiNb_2_O_7_, Ti_2_Nb_10_O_29_, Ti_2_Nb_14_O_39_, and TiNb_24_O_62_ electrodes are 0.31, 0.42, 0.20, and 0.87 respectively (Fig. S5†). Note that Nb/Ti ratios in TiNb_2_O_7_, Ti_2_Nb_10_O_29_, Ti_2_Nb_14_O_39_, and TiNb_24_O_62_ are 2, 5, 7, and 24 respectively.

Fig. 4c and d show the radial structure function of TNO samples obtained using Fourier transforms of the Nb K-edge EXAFS (extended X-ray absorption fine structure) oscillations at fully lithiated and delithiated states. The continuous spectral changes are provided in Fig. S6.† Because the EXAFS data are qualitative, we focused on identifying the differences between TNO compositions and distinct properties from previously reported Nb_2_O_5_ anodes.^31^ The peaks at around 1.7–2.2 Å in Fig. 4d correspond to the Nb–O interaction and the peaks at around 2.8–3.5 Å in Fig. 4d correspond to the Nb–TM (transition metal) interaction. In all samples, the peaks of Nb–O interaction are characteristically higher than those of Nb–TM interaction in the fully lithiated state, and in contrast, the peaks of Nb–TM interaction are higher than those of Nb–O interaction in the fully delithiated state. This phenomenon corresponds to the lithium-ion diffusion mechanism in the tetragonal Nb_2_O_5_ anode.^31^ This suggests that Li ions diffuse through the Nb–TM interlayer in TNO anodes similar to lithium-ion diffusion through the Nb–Nb interlayer in tetragonal Nb_2_O_5_ anodes.^31^ This is evidence that TiNb_2_O_7_, which has a higher O/Nb ratio compared to the other TNO samples, can be advantageous in lithium-ion diffusion. In other words, the lower O/Nb ratio in TNO anodes, the lower the rate performance.

**Fig. 5:**
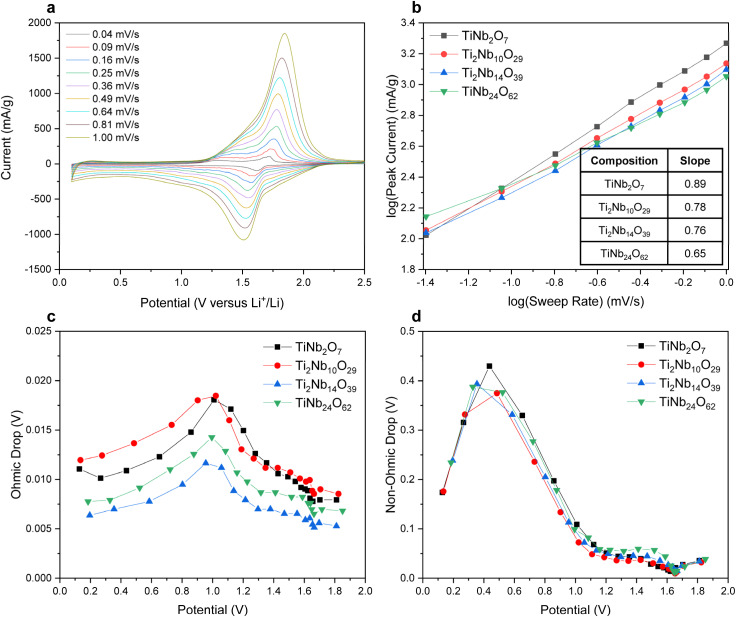
Electrochemical analysis of the TNO anodes. (a) CV results of the TiNb_2_O_7_ anode (scan rate: 0.04–1.00 mV s^−1^), (b) log(sweep rate) *versus* log(peak current) graph from the CV results, (c) ohmic overpotentials derived using the GITT results and (d) non-ohmic overpotentials derived using the GITT results.

To verify this trend, we measured the diffusion properties of our different TNO anodes using a CV based method published previously.^18,32^Fig. 5a shows the CV data for TiNb_2_O_7_ and those of the other TNO anodes are shown in Fig. S7.† We plotted the log(sweep rate) *versus* log(peak current) graph to investigate the redox mechanism of TNO anodes (Fig. 5b). In this graph, if the slope is close to 1, there is no diffusion limit (capacitive behaviour), and if it is close to 0.5, it has general diffusion properties.^18,32^ As shown in Fig. 5b, the slope values of TiNb_2_O_7_, Ti_2_Nb_10_O_29_, Ti_2_Nb_14_O_39_, and TiNb_24_O_62_ electrodes are 0.89, 0.78, 0.76, and 0.65 respectively. The slope was the highest in the TiNb_2_O_7_ anode and is decreasing with the oxygen to Nb and Ti ratio. This confirms the trend measured by EXAFS, which suggests faster kinetics in TiNb_2_O_7_. Next, the GITT (Galvanostatic Intermittent Titration Technique) was conducted to quantify both ohmic and non-ohmic overpotentials in different TNO compositions as a function of the state of charge (during lithiation) (see the ESI† for a detailed pulse method). The raw data of GITT data are shown in Fig. S8.†Fig. 5c shows that the ohmic overpotentials range from 0.005–0.020 V. The non-ohmic drops are placed in the range of 0.00–0.45 V (Fig. 5d). The overpotential difference between samples was smaller than the overpotential difference according to SOC. Therefore, the rate performance of TNO anodes is determined more by diffusion than by the difference in overpotential.

## Conclusions

In conclusion, we synthesised four classes of TNO anodes with different Nb and Ti contents (TiNb_2_O_7_, Ti_2_Nb_10_O_29_, Ti_2_Nb_14_O_39_, and TiNb_24_O_62_) using the same solid state synthesis method to obtain materials that can be compared directly. The different Ti and Nb compositions result in different electrochemical performances. Among them, TiNb_2_O_7_ showed the lowest gravimetric capacity but the best cycling and rate performance. To understand these differences in performance, we measured the changes in the oxidation state of Nb *via operando* XAS and calculated the changes in the oxidation state of Ti. Interestingly, the oxidation state change of Nb in TiNb_2_O_7_ was greater than that in our other TNO anodes. In other words, the TiNb_2_O_7_ anode used the oxidation state of Nb more efficiently and relied less on changes in the oxidation state of Ti. The increase in the Ti to Nb ratio in the TNO anode seems to lead to increases in gravimetric capacity, but at the cost of decreased cycling stability, providing new guidelines for material design. Similarly, we observed that the relative oxygen content in TNO anodes affects performance, where a higher O/Nb ratio demonstrated superior rate performance.

## Data availability

The raw data supporting the findings of this study are available at the University of Cambridge's open data repository under https://doi.org/10.17863/CAM.116030.

## Conflicts of interest

There are no conflicts to declare.

## Supplementary Material

TA-013-D4TA08141B-s001
