# Supplementary material for: Compositional study of Ti–Nb oxide (TiNb2O7, Ti2Nb10O29, Ti2Nb14O39, and TiNb24O62) anodes for high power Li ion batteries
Source: J Mater Chem A Mater. 2025 Feb 19;13(14):9878–85. doi: 10.1039/d4ta08141b (PMC11891643; doi:10.1039/d4ta08141b)
Supplement: TA-013-D4TA08141B-s001 [file TA-013-D4TA08141B-s001.pdf]

This journal is © The Royal Society of Chemistry 2025

Supplementary Information

Compositional Study of Ti-Nb Oxide ($TiNb_2O_7$, $Ti_2Nb_{10}O_{29}$, $Ti_2Nb_{14}O_{39}$, and $TiNb_{24}O_{62}$) Anode for High Power Li Ion Batteries

Yeonguk Son[a,b]†, Haeseong Jang[c]†, Bo Wen[a], Changshin Jo[d], Alexander S Groombridge[e], Adam Boies[f], Min-Gyu Kim[g]*, Michael De Volder[a]*

[a]Department of Engineering, University of Cambridge, 17 Charles Babbage Road, CB3 0FS Cambridge, UK. E-mail: mfld2@cam.ac.uk

[b]Department of Chemical Engineering, Changwon National University, Changwon, Gyeongsangnam-do 51140, Republic of Korea

[c]Department of Advanced Materials Engineering, Chung-Ang University, 4726, Seodong-daero, Daedeok-myeon, Anseong, Gyeonggi-do 17546, the Republic of Korea

[d]Graduate Institute of Ferrous and Energy Materials Technology (GIFT), Pohang University of Science and Technology (POSTECH), 77 Cheongam-Ro, Nam-Gu, Pohang, Gyeongbuk, 37673, Republic of Korea

[e]Echion Technologies Ltd, Unit 9, Cambridge South, West Way, CB22 3FG Cambridge, UK

[f]Department of Mechanical Engineering, Stanford University, Stanford, CA 94305

[g]Beamline Research Division, Pohang Accelerator Laboratory (PAL), Pohang 37673, Republic of Korea. E-mail: habga82@postech.ac.kr

† These authors contributed equally to this work.

Experimental section

Preparation of Ti-Nb oxide

To make a homogeneous mixture of TiO_2 (Anatase, nanopowder, <25 nm, Sigma-Aldrich) and NbO_2 (Sigma-Aldrich) precursors, a blade blender was utilized for 1-2 minutes. Depending on the compositional ratio of products ($TiNb_2O_7$, $Ti_2Nb_{10}O_{29}$, $Ti_2Nb_{14}O_{39}$, and $TiNb_{24}O_{62}$), the molar ratio of TiO_2 and NbO_2 precursors was controlled. For example, 7.50 g (0.0939 mol) of TiO_2 and 23.46 g (0.1878 mol) of NbO_2 were mixed to synthesize the $TiNb_2O_7$. Collected powder was heated in a tube furnace under oxygen atmosphere. First, heating temperature was raised to 450 °C with ramping rate of 1 °C min^{-1} and duration time for 5 hours, an then it is raised to 1000 °C with ramping rate of 1 °C min^{-1} and duration time for 10 hours.

Materials characterization

The morphology of the samples was measured using scanning electron microscopy (SEM, Leo Variable pressure SEM with an acceleration voltage of 10 kV). PXRD spectra were collected on a Panalytical Empyrean diffractometer (Cu Kα1=1.5405 Å, Kα2=1.5443 Å) under room temperature in Bragg-Brentano geometry. The powder samples are packed on a flat circular quartz holder and scanned in a 2θ range of 5-90° at a rate of circa. 1.42°/min. The sample stage was set to rotate continuously to improve powder averaging. The particle size distribution was measured using laser diffraction particle size analyser (Microtrac S3500, Microtrac).

Rietveld refinement

The diffraction patterns are refined using GSAS-II. Chebyshev background coefficients, lattice parameters, sample displacement, crystallite size, micro-strain and other peak profile parameters were refined. In this case, atomic coordinates were not refined due to the large number of variables this may introduce and the weak relative scattering of oxygen compared to titanium and niobium.

Operando XAS

X-ray absorption spectroscopy (XAS), including X-ray absorption near edge structure (XANES) and extended X-ray absorption fine structure (EXAFS), was conducted on the Wide Energy XAFS beam

line (multipole-wiggler BL10C beam line) at the Pohang Light Source-II (PLS-II) with top-up mode operation under a ring current of 250 mA at 3.0 GeV. The incident beam from multipole-wiggler source was monochromatized by using a liquid nitrogen-cooled double-crystal monochromator (Bruker ASC). The incident and transmitted X-ray photon counts were measured by using nitrogen gas-filled ionization chambers (IC-SPEC, FMB Oxford). In the case of Nb K-edge XAS, Si(111) crystal pair double-crystal monochromator and transmittance mode was used. The intensity of incident X-ray beam was detuned to ~80 % in order to eliminate higher-order harmonic effects. Energy calibration processes were carried out simultaneously for each measurement with reference metal foil (Nb metal). For operando XAS analysis, home-made electrochemicl puch-type half-cell, which consist of sample loaded electrode, separator, lithium metal, and electrolyte, was employed. The operando XAS spectra for Nb K-edge were collected with ~25 minutes scanning time for one spectra, conducting 0.2 C-rate charging and discharging process. The acquired spectra were converted into normalized XANES and Fourier-transformed radial distribution functions (RDFs) by Athena software based on standard XAFS procedure. The oxidation states of Nb were obtained from least-square method (LSM).

The photon energy corresponding to the Ti K-edge is almost entirely absorbed by the thick Cu foil and therefore the oxidation state cannot be measured directly by operando XANES. To address this limitation, we calculated changes in the oxidation state of Ti indirectly by measuring changes in the oxidation state of Nb using XANES, along with the capacity. For instance, the reversible capacity of the $TiNb_2O_7$ anode was measured to be 278 mAh/g, which corresponds to 0.01037 mol/g of electrons (or Li ions) as determined by the following equation:

$$0.278 Ah/g \times \frac{3600\ s}{1\ hour} \times \frac{1}{96500 C/mol} = 0.010371\ mol/g$$

Given the molecular weight of $TiNb_2O_7$ is 345.69 g/mol, the stoichiometric quantity of Li ions, x, in the reaction

$$x\mathrm{Li}^+ + x\mathrm{e}^- + \mathrm{TiNb_2O_7} \rightarrow \mathrm{LiTiNb_2O_7}$$

is calculated to be 3.59. Based on XANES analysis, the oxidation state change of Nb was determined to be 1.64. Consequently, the oxidation state change of Ti was calculated to be 0.31 using the equation $3.59-2\times1.64=0.31$.

Electrochemical characterization

To prepare the electrode, TNO anodes material ($TiNb_2O_7$, $Ti_2Nb_{10}O_{29}$, $Ti_2Nb_{14}O_{39}$, or $TiNb_{24}O_{62}$) was mixed with CMC, SBR, and super-P at a mass ratio of 80: 5: 5: 10 in distilled water. The doctor blade gap was adjusted to achieve loading level of 1.5~2.0 mg/cm2. The electrodes were calendered to

achieve the electrode density of 1.5~2.0 g/cm3. The electrodes were vacuum dried at 120 ℃ for 6 hours before coin cell assembly. Cut-off voltage of half cell was 0.01-2.5 V. C-rate of formation 2 cycles was 0.05C (1C = 250 mA/g). For the cycling test, 0.5C was used. For the rate test in half cell, charging/discharging rate range of 0.5, 1, 2, 5, 10, and again 0.5C was used for each 5 cycles. For the GITT test, the pulse current was 0.1C for 30 minutes, followed by 2 hours rest time. Cut-off voltage of full cell was 3.0-1.0 V. C-rate of formation 2 cycles was 0.05C. For the cycling test, 0.5C was used. For the rate test in full cell, 0.5C charging rate was fixed and discharging rate range of 0.5, 1, 2, 3, 4, 5C was used for each 5 cycles. For the CV test, scan rate range was 0.04~1.0 mV/s (0.04, 0.09, 0.16,0.25, 0.36, 0.49, 0.64, 0.81, and 1 mV/s).

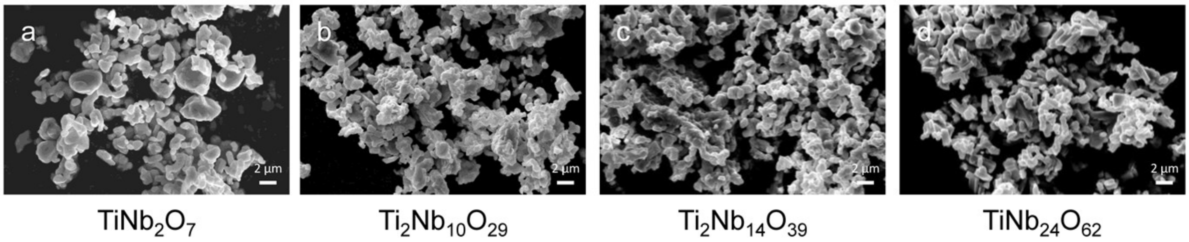

Figure S1. SEM images of (a) $TiNb_2O_7$, (b) $Ti_2Nb_{10}O_{29}$, (c) $Ti_2Nb_{14}O_{39}$, and (d) $TiNb_{24}O_{62}$

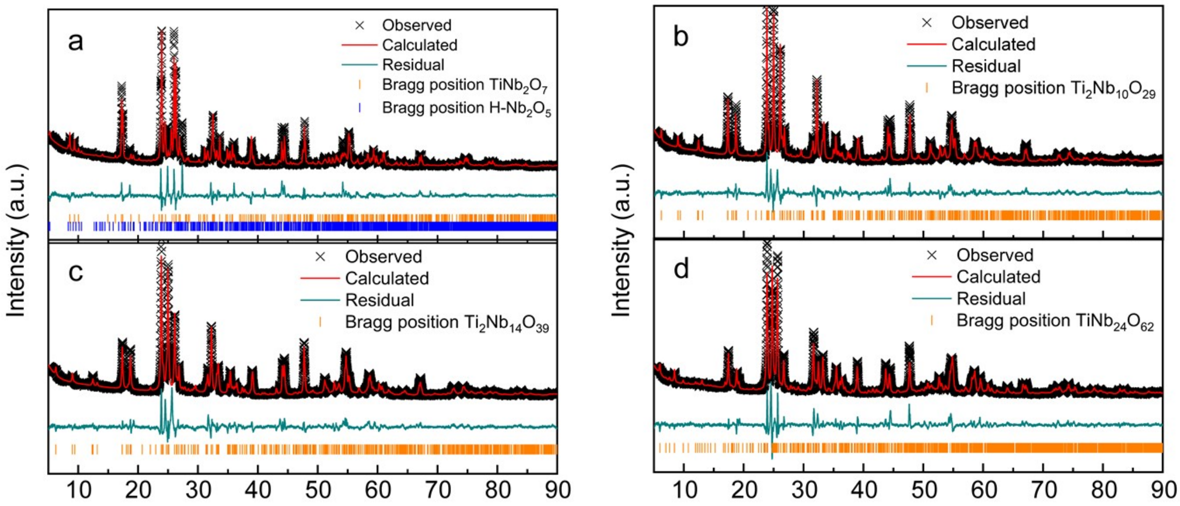

Figure S2. Rietveld refinement results of (a) $TiNb_2O_7$, (b) $Ti_2Nb_{10}O_{29}$, (c) $Ti_2Nb_{14}O_{39}$, and (d) $TiNb_{24}O_{62}$

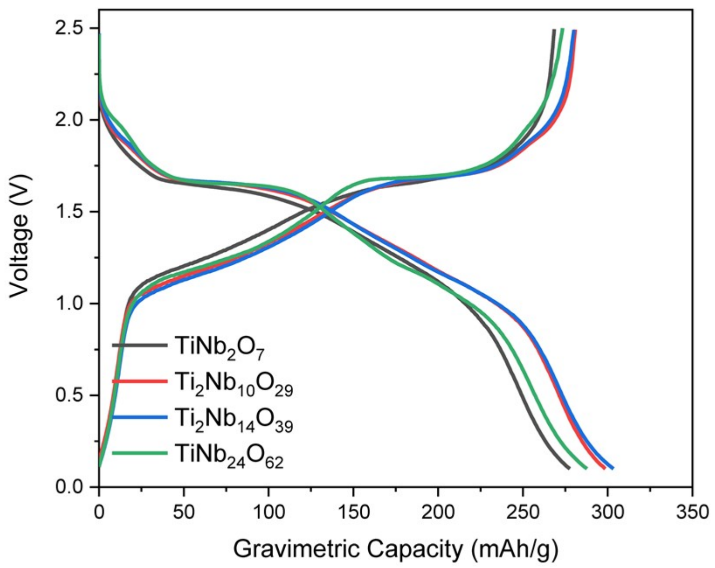

Figure S3. 0.05C second formation voltage profiles of the TNO anodes

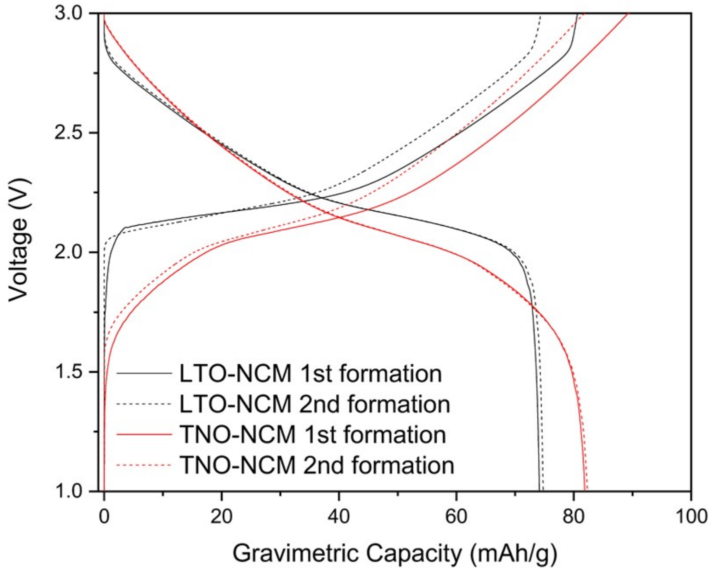

Figure S4. full cell formation voltage profiles of LTO-NCM and TNO-NCM full cells.

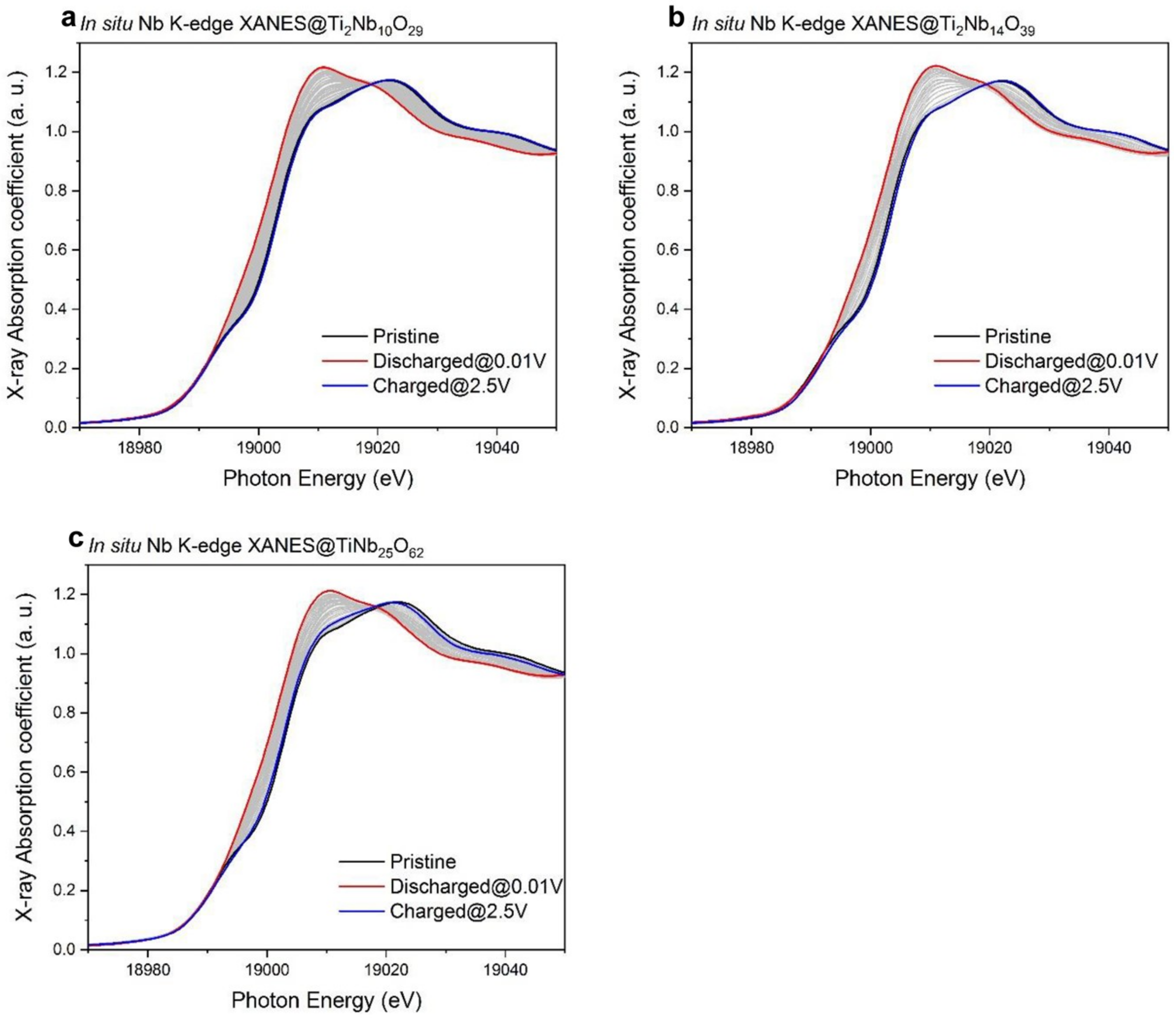

Figure S5. the Nb K-edge XANES absorption spectra of (a) $Ti_2Nb_{10}O_{29}$, (b) $Ti_2Nb_{14}O_{39}$, and (c) $TiNb_{24}O_{62}$ anodes at fully lithiated and delithiated states

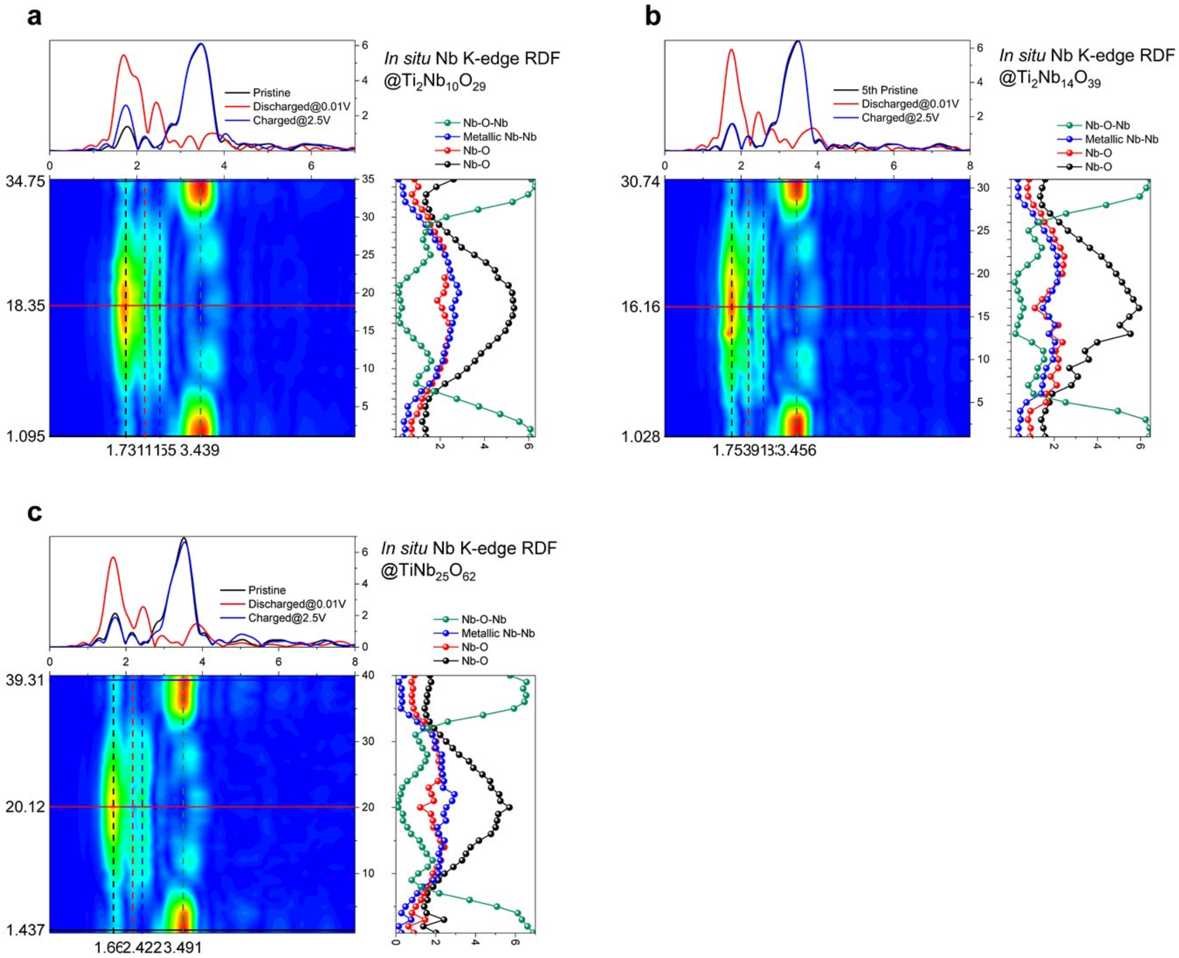

Figure S6. EXAFS results of (a) $Ti_2Nb_{10}O_{29}$, (b) $Ti_2Nb_{14}O_{39}$, and (c) $TiNb_{24}O_{62}$ anodes

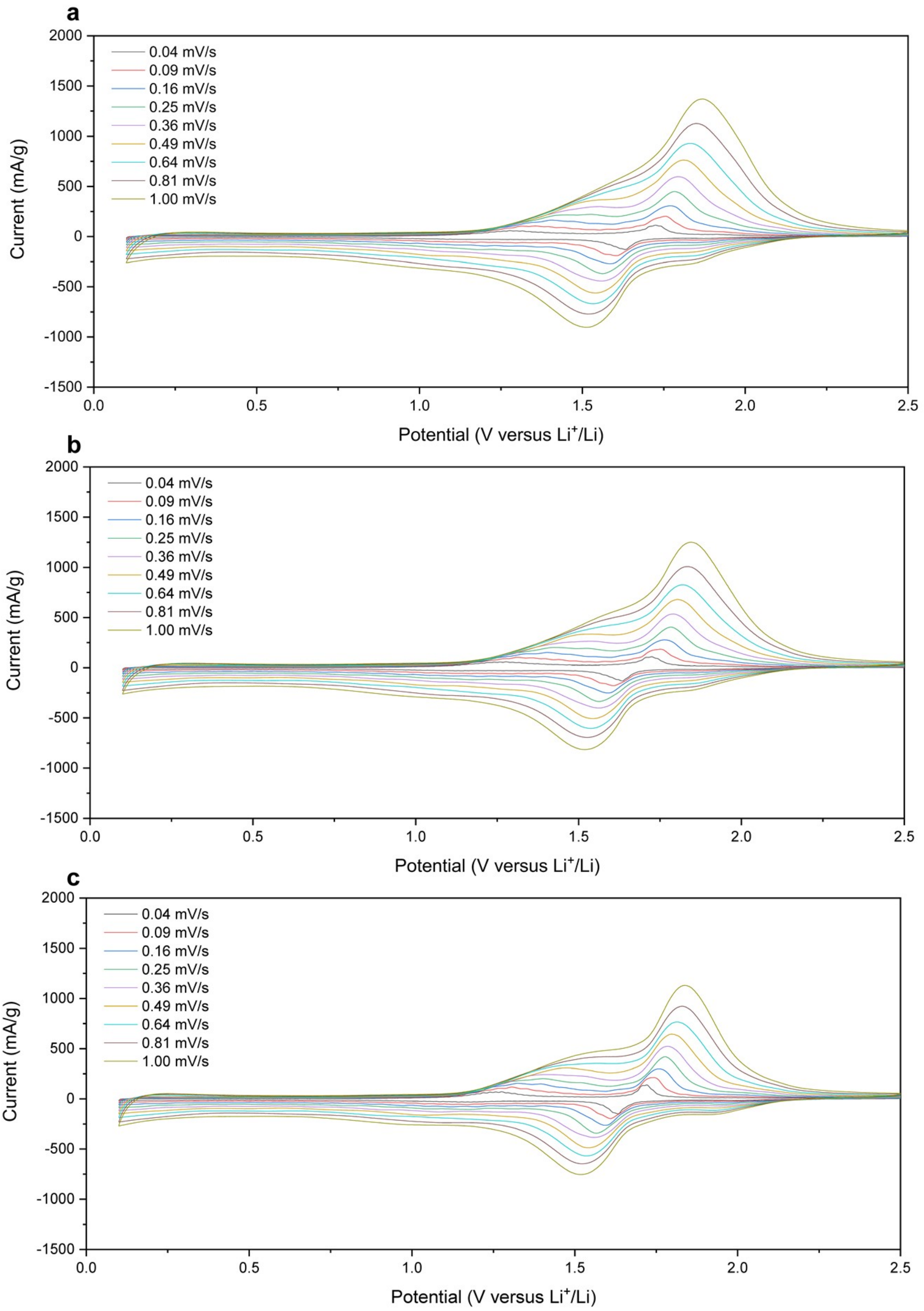

F

igure S7. CV results of (a) $Ti_2Nb_{10}O_{29}$, (b) $Ti_2Nb_{14}O_{39}$, and (c) $TiNb_{24}O_{62}$ anodes (scan rate: 0.04~1.00 mV/s)

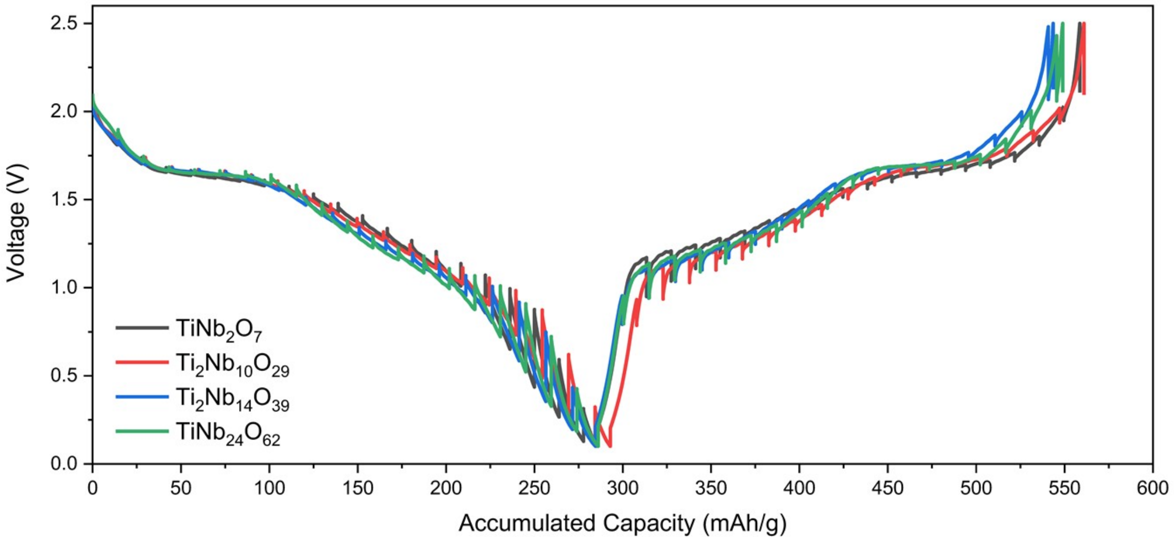

Figure S8. GITT raw data of TNO anodes

Sample	a (Å)	b (Å)	c (Å)	α, γ (°)	β (°)	V (Å^3)	R_{wp} (%)	GOF
$TiNb_2O_7$	20.375(5)	3.80104(9)	11.894(2)	90	120.220(5)	795.97(3)	9.38	3.00
$Ti_2Nb_{10}O_{29}$	15.518(1)	3.81122(8)	20.545(3)	90	113.031(4)	1118.23(4)	8.43	2.80
$Ti_2Nb_{14}O_{39}$	15.535(2)	3.81387(11)	20.556(4)	90	113.074(6)	1120.48(6)	9.60	3.20
$TiNb_{24}O_{62}$	29.782(1)	3.81335(12)	21.104(1)	90	95.043(5)	2387.52(2)	9.63	3.18

Table S1. Results of crystal analysis by Rietveld refinements in different compositions.

Sample	Cut-off Voltage	1st Delithiation (mAh/g)	1st Coulombic Efficiency (%)	Reference
$TiNb_2O_7$	0.8-3.0 V	281 mAh/g	93%	[27]
$TiNb_2O_7$	1.0-3.0 V	210 mAh/g	84%	[14]
$TiNb_2O_7$ with partial nitridation	1.0-3.0 V	265 mAh/g	92%	[6]
$Ti_2Nb_{10}O_{29}$	1.0-2.4 V	293 mAh/g	94%	[16]
$TiNb_2O_7$ with $Ti_{1-x}Nb_xN$	1.0-3.0 V	254 mAh/g	94%	[24]
$Ti_2Nb_{10}O_{29}$ with RGO	1.0-2.5 V	256 mAh/g	98%	[31]
$Ti_2Nb_{10}O_{29}$	0.8-2.5 V	312 mAh/g	91%	[5]
$TiNb_{24}O_{62}$	1.0-3.0 V	240 mAh/g	89%	[19]
$Ti_{0.98}Nb_{2.02}O_7$	1.0-3.0 V	260 mAh/g	> 92%	[10]
Ti-Nb oxide	1.0-2.5 V	254 mAh/g	97%	[23]
$Ti_2Nb_{14}O_{39}$	0.8-3.0 V	326 mAh/g	88%	[22]
$TiNb_2O_7$	1.0-2.5 V	260 mAh/g	97%	[17]

Table S2. Summary of previous publications regarding TNO anodes
